# Polyelectrolyte Complexes between Polycarboxylates and BMP-2 for Enhancing Osteogenic Differentiation: Effect of Chemical Structure of Polycarboxylates

**DOI:** 10.3390/polym11081327

**Published:** 2019-08-09

**Authors:** Masahiko Terauchi, Atsushi Tamura, Asato Tonegawa, Satoshi Yamaguchi, Tetsuya Yoda, Nobuhiko Yui

**Affiliations:** 1 Department of Maxillofacial Surgery, Graduate School of Medical and Dental Science, Tokyo Medical and Dental University (TMDU), 1-5-45 Yushima, Bunkyo, Tokyo 113-8549, Japan; 2 Department of Organic Biomaterials, Institute of Biomaterials and Bioengineering, Tokyo Medical and Dental University (TMDU), 2-3-10 Kanda-Surugadai, Chiyoda, Tokyo 101-0062, Japan

**Keywords:** bone morphogenetic protein-2, osteogenic differentiation, polyelectrolyte complex, polycarboxylate, poly(glutamic acid)

## Abstract

Bone morphogenetic protein 2 (BMP-2) has received considerable attention because of its osteoinductivity, but its use is limited owing to its instability and adverse effects. To reduce the dose of BMP-2, complexation with heparin is a promising approach, because heparin enhances the osteoinductivity of BMP-2. However, the clinical use of heparin is restricted because of its anticoagulant activity. Herein, to explore alternative polymers that show heparin-like activity, four polycarboxylates, poly(acrylic acid) (PAA), poly(methacrylic acid) (PMAA), poly(aspartic acid) (PAsp), and poly(glutamic acid) (PGlu), were selected and their capability to modulate the osteoinductivity of BMP-2 was evaluated. Dynamic light scattering indicated that these polycarboxylates formed polyelectrolyte complexes with BMP-2. The osteogenic differentiation efficiency of MC3T3-E1 cells treated with the polycarboxylate/BMP-2 complexes was investigated in comparison to that of the heparin/BMP-2 complex. As a result, PGlu/BMP-2 complex showed the highest activity of alkaline phosphatase, which is an early-stage marker of osteogenic differentiation, and rapid mineralization. Based on these observations, PGlu could serve as an alternative to heparin in the regenerative therapy of bone using BMP-2.

## 1. Introduction

Autologous bone grafts are widely used in the clinical treatment of bone defects in maxillofacial and plastic surgery [1,2,3]. Although this approach has been recognized as a gold standard for bone reconstruction, the collection of bone grafts is generally invasive for patients. As an alternative method to bone grafting, various approaches to stimulate bone regeneration have been investigated, such as artificial bone substitute materials (e.g., β-tricalcium phosphate and hydroxyapatite) [4,5] and pharmacological molecules (e.g., growth factors and low-molecular-weight drugs) [6,7,8,9,10]. Bone morphogenetic protein 2 (BMP-2), a secreted growth factor that belongs to the transforming growth factor-β (TGF-β) superfamily, has attracted considerable attention because of its strong osteoinductivity [11,12]. In recent years, BMP-2 has been applied for the treatment of spine fusion, bone fracture, and periodontal tissue therapy [13,14,15], and it is expected that clinical application will continue to expand. However, BMP-2 is easily deactivated under physiological conditions. In other words, high doses of BMP-2 are required to maintain its activity in the long term and to regenerate a wide range of bone defects. Unfortunately, it is reported that BMP-2 at high doses possibly induces adverse effects, such as inflammation at the implantation site, bone resorption by increasing osteoclastic activity, and the induction of structurally abnormal bone [16,17,18]. To reduce the dose of BMP-2 while maintaining its activity, heparin has received much attention because the mixture of BMP-2 and heparin is known to enhance osteoinductivity [19,20,21,22,23]. The negatively charged heparin forms a polyelectrolyte complex with the positively charged BMP-2 (isoelectric point: 8.5), protecting BMP-2 from deactivation and enhancing osteoinductivity of BMP-2 [24]. However, the clinical use of heparin might be limited because of its strong anticoagulant effect [25,26].

**Figure 1:**
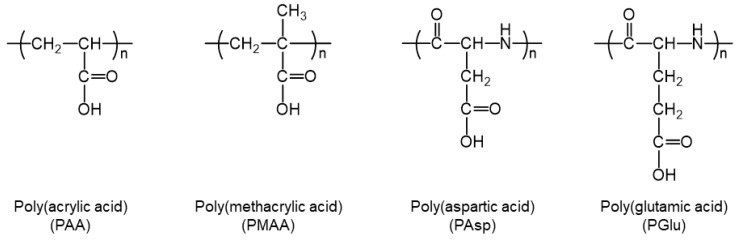
Chemical structures of polycarboxylates, where *n* indicates the degree of polymerization.

As an alternative to heparin, various sulfated and sulfonated polymers have been investigated to enhance the osteoinductivity of BMP-2 without anticoagulant activity and toxicity. For example, our group has developed sulfonated polyrotaxanes that formed polyelectrolyte complexes with BMP-2, which enhanced osteogenic differentiation efficiency and promoted bone regeneration in a mouse model of calvarial defect [6,7]. Liu and co-workers developed BMP-2-loaded 2-*N*,6-*O*-sulfated chitosan nanoparticles and investigated their capability of bone regeneration in a rabbit critical-sized radial defect [27,28]. The effect of sulfated and sulfonated polymers in modulating the activity of BMP-2 has also been observed for other growth factors such as basic fibroblast growth factor (bFGF). Maynard and co-workers investigated the heparin-mimicking effect of synthetic polymers and identified that poly(vinyl sulfonate) had the strongest ability to enhance the binding of bFGF to FGF receptor [29]. Although heparin is utilized for surface modification, conjugation of functional molecules, and preparation of microparticles to fabricate bone regenerative biomaterials [30,31,32], it is difficult to apply sulfated and sulfonated polymers as a building block of biomaterials owing to the poor reactivity of sulfate and sulfonate groups. On the other hand, carboxy groups are also negatively charged and easily react with amino and hydroxy groups in the presence of a condensation reagent. Therefore, negatively charged polycarboxylates offer an appealing property for the fabrication of biomaterials. Sulfated and sulfonated polymers have been widely investigated for the modulation of growth factor activity, but negligible studies have been reported on polycarboxylates. Herein, we demonstrated the effect of a series of four polycarboxylates, poly(acrylic acid) (PAA), poly(methacrylic acid) (PMAA), poly(aspartic acid) (PAsp), and poly(glutamic acid) (PGlu) (Figure 1), in enhancing the osteogenic differentiation efficiency of BMP-2.

## 2. Materials and Methods

### 2.1. Materials

Recombinant human BMP-2 was obtained from R&D Systems (Minneapolis, MN, USA). PAA (catalog molecular weight: 5000) was obtained from Fujifilm Wako Pure Chemical Corporation (Osaka, Japan). Heparin, PMAA (catalog molecular weight: 7750), PAsp (poly(α,β-dl-aspartic acid); catalog molecular weight: 2000 to 11,000), and PGlu (poly(l-glutamic acid); catalog molecular weight: 1500 to 5500) were obtained from Sigma-Aldrich (Milwaukee, WI, USA).

### 2.2. Size Exclusion Chromatography (SEC)

SEC was performed using a high-performance liquid chromatography system consisting of an AS-2057i Plus autosampler (Jasco, Tokyo, Japan), DG-2080-53 degasser (Jasco), PU-2080i Plus pump (Jasco), CO-965 column oven (Jasco), RI-2031 Plus refractive index detector (Jasco), and a combination of TSKgel G4000PW_XL_ and G2500PW_XL_ columns (300 mm × 7.8 mm internal diameter) (Tosoh, Tokyo, Japan). The sample solutions (10 mg/mL, 50 μL) were injected into the system and eluted with 100 mM NaNO_3_ solution at a flow rate of 1 mL/min at 40 °C. The relative number-average molecular weight (*M*_n,SEC_) and molecular weight distribution (*M*_w_/*M*_n_) were calculated from a calibration curve of standard poly(ethylene glycol) (PEG; Agilent Technologies, Wilmington, DE, USA).

### 2.3. Potentiometric Titration

The polycarboxylates were dissolved in 7.5 mM NaOH solution (20 mL) at a carboxy concentration of 5 mM. The solutions were titrated with 10 mM HCl at 25 ± 1 °C using an automatic titrator (AUT-701; DKK-TOA, Tokyo, Japan). The titrant was added in quantities of 50 μL at an interval of 10 s. The p*K*_a_ and degree of protonation (α) were calculated from the pH-α curves.

### 2.4. Dynamic Light Scattering (DLS) of the Polycarboxylate/BMP-2 Complexes

BMP-2 was dissolved in 10 mM phosphate buffer at a concentration of 200 μg/mL. Separately, the polycarboxylates were dissolved in 10 mM phosphate buffer at a concentration 2 mg/mL. BMP-2 and the polycarboxylate solutions were combined at an equal volume ratio to form the polyelectrolyte complexes. The final concentrations of BMP-2 and the polycarboxylates were 100 μg/mL and 1 mg/mL, respectively. DLS measurements were performed on a Zetasizer Nano ZS (Malvern Instruments, Malvern, UK) equipped with a 4 mW He-Ne Laser (633 nm). The measurements were conducted at 25.0 °C at a detection angle of 173°.

### 2.5. Cell Culture

MC3T3-E1 cells subclone 4, an osteoblastic cell line derived from mouse calvaria, was obtained from the American Type Culture Collection (ATCC; Manassas, VA, USA). The MC3T3-E1 cells were cultured in α-minimum essential medium (α-MEM; Fujifilm Wako Pure Chemical) supplemented with 10% fetal bovine serum (FBS; Gibco, Grand Island, NY, USA), 1% penicillin (100 U/mL; Fujifilm Wako Pure Chemical), streptomycin (100 μg/mL; Fujifilm Wako Pure Chemical), and l-alanyl-l-glutamine (2 mM; Fujifilm Wako Pure Chemical) in 5% CO_2_ at 37 °C.

### 2.6. Cytotoxicity of Polycarboxylates

MC3T3-E1 cells were plated in 96-well plates at a density of 1 × 10^4^ cells/well and incubated for 24 h. The medium was replaced with treatment medium (100 μL) containing the polycarboxylates and heparin (0.005 to 500 μg/mL). After 24 h of incubation, Cell Counting Kit-8 reagent (Dojindo Laboratories, Kumamoto, Japan) was added to each well (10 μL/well) and incubated for 1 h at 37 °C. The absorbance at 450 nm was measured on a Multiskan FC plate reader (Thermo Fisher Scientific, Waltham, MA, USA). Cellular viability was calculated relative to that of untreated cells.

### 2.7. Anticoagulant Activity of Polycarboxylates

The anticoagulant activity of the polycarboxylates and heparin was evaluated using Test Team Heparin S (Sekisui Medical, Tokyo, Japan) according to the manufacturer’s instructions. Each polymer solution (32 μL) was combined with plasma (4 μL) and anti-thrombin III solution (4 μL) in 96-well plates (Thermo Fisher Scientific) and incubated for 5 min at 37 °C. Factor Xa solution (20 μL) was then added to each well and incubated for 30 s at 37 °C. A chromogenic substrate solution (40 μL; substrate: S-2222) was added to each well and incubated for 3 min at 37 °C. Finally, the absorbance at 405 nm was measured on a Multiskan FC plate reader.

### 2.8. Osteogenic Differentiation and Alkaline Phosphatase (ALP) Activity in MC3T3-E1 Cells

MC3T3-E1 cells were plated in a 24-well plate at a density of 1 × 10^5^ cells/well and incubated for 24 h. The medium was replaced with osteogenic differentiation medium (300 μL) supplemented with 10% FBS, ascorbic acid (50 μg/mL; Sigma-Aldrich), β-glycerophosphate disodium salt (10 mM; Sigma-Aldrich), and each BMP-2 complex (50 ng/mL BMP-2 and 500 μg/mL polymers). After 72 h of incubation, the cells were washed twice with phosphate-buffered saline (PBS). The cell lysate was prepared using radioimmunoprecipitation assay (RIPA) buffer (Fujifilm Wako Pure Chemical) containing a Complete Protease Inhibitor Cocktail (Roche, Basel, Switzerland). The enzymatic activity of ALP in the lysate was determined using LabAssay ALP (Fujifilm Wako Pure Chemical) according to the manufacturer’s instructions. The absorbance at 405 nm was measured on a Multiskan FC plate reader. The concentration of total protein in the cell lysates was determined using a Micro BCA Protein Assay Kit (Thermo Fischer Scientific) according to the manufacturer’s instruction. The activity of ALP was normalized to the amount of protein.

### 2.9. Mineralization of MC3T3-E1 Cells

MC3T3-E1 cells were cultured in osteogenic differentiation medium as described above. The concentrations of BMP-2 and the polymers were 100 ng/mL and 1,000 μg/mL, respectively. The medium was changed twice every week. After 14 days of incubation, the cells were fixed with methanol for 10 min, followed by staining with 1% Alizarin red S solution for 5 min. After the cells were washed with PBS, images of cells were acquired using an IX-71 microscope (Olympus, Tokyo, Japan) equipped with a DP-80 dual charge-coupled device (CCD) microscope camera (Olympus). The stained areas in the images were quantified using Image J software ver. 1.45l (National Institutes of Health, Bethesda, MD, USA).

### 2.10. Statistical Analysis

Data were analyzed by one-way analysis of variance (ANOVA) followed by Tukey–Kramer multiple comparison test. A *p* value of less than 0.05 was considered to indicate statistical significance.

## 3. Results and Discussion

### 3.1. Chemical Characterizations of Polycarboxylates

**Figure 2:**
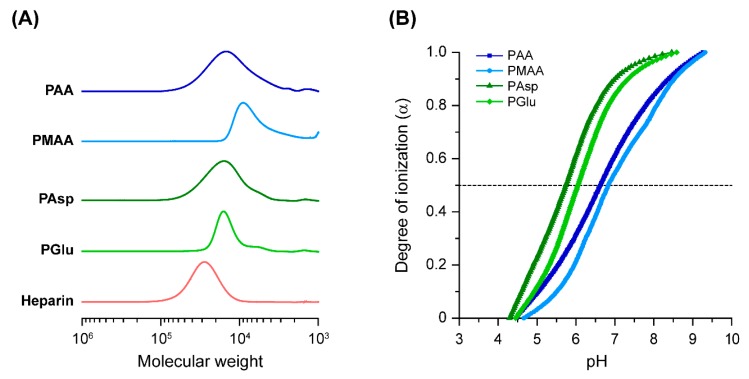
(**A**) Size exclusion chromatography (SEC) charts of polycarboxylates and heparin eluted with 100 mM NaNO_3_ solution at 40 °C. (**B**) pH-α curves of polycarboxylates at 25 ± 1 °C. The plots represent poly(acrylic acid) (PAA, squares), poly(methacrylic acid) (PMAA, circles), poly(aspartic acid) (PAsp, triangles), and poly(glutamic acid) (PGlu, diamonds).

In this study, the capability of four commercially available polycarboxylates to enhance osteogenic differentiation efficiency of BMP-2 was assessed. Although the molecular weights of these polycarboxylates were provided by the supplier, their methods of determination were different. Additionally, while the p*K*_a_ values of the polycarboxylates have been previously reported in the literature, the experimental conditions to determine the p*K*_a_ values were not identical. Therefore, the relative number-average molecular weight (*M*_n,SEC_) and p*K*_a_ of the polycarboxylates were determined by SEC and potentiometric titration, respectively. Figure 2A shows the SEC charts of the polycarboxylates and heparin in 100 mM NaNO_3_ solution. From these results, the *M*_n,SEC_ and *M*_w_/*M*_n_ of the polycarboxylates and heparin were calculated based on the calibration of PEG (Table 1). The *M_n_*_,SEC_ of the polycarboxylates ranged from 5100 to 13,500 with relatively broad molecular weight distribution. Although the molecular weight of the polycarboxylates were not identical, we utilized these polymers for further studies. The p*K*_a_ values and degree of ionization (α) at pH 7.4 were determined from the pH-α curves (Figure 2B and Table 1). The p*K*_a_ values of the (meth)acrylate-based polymers (PAA and PMAA) were slightly higher than the polyamino acids (PAsp and PGlu). The α values of PAsp and PGlu at pH 7.4 were over 0.9, which were higher than those of PAA and PMAA (0.64 to 0.71). It is considered that the difference in main chain structure might change the p*K*_a_ and α.

**Figure 3:**
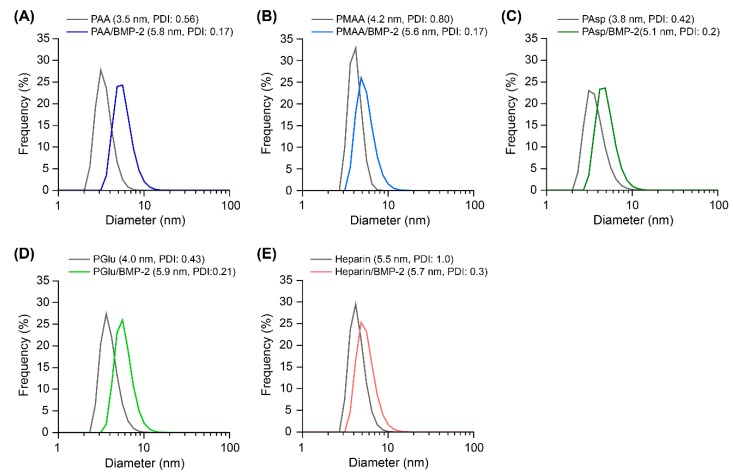
Size distributions of the polymers and their complexes with bone morphogenetic protein 2 (BMP-2): (**A**) PAA and PAA/BMP-2, (**B**) PMAA and PMAA/BMP-2, (**C**) PAsp and PAsp/BMP-2, (**D**) PGlu and PGlu/BMP-2, and (**E**) heparin and heparin/BMP-2 in 10 mM phosphate buffer at pH 7.4. The concentrations of BMP-2 and polymers were 100 μg/mL and 1 mg/mL, respectively.

Next, the polyelectrolyte complex formation of BMP-2 with polycarboxylates was investigated using DLS (Figure 3). The polyelectrolyte complexes between polycarboxylates and BMP-2 were prepared in 10 mM phosphate buffer at pH 7.4. The size distributions of the polymer/BMP-2 complexes were approximately 5 to 6 nm, which were slightly shifted to large size and narrow polydispersity index (PDI) in comparison to the polymers for all polycarboxylates and heparin. From the size of the complexes, it is suggested that a few polymer chains covered the surface of BMP-2. Aggregation was not observed probably because of the excess of polymers. Additionally, the difference in the α of polycarboxylates was not reflected in the size of the complexes. In our previous study on the polyelectrolyte complexes of sulfonated polyrotaxanes and BMP-2, similar tendencies were observed [7]. Accordingly, it is considered that the polycarboxylates and heparin formed a polyelectrolyte complex with BMP-2.

### 3.2. Biological Characterizations of Polycarboxylates

**Figure 4:**
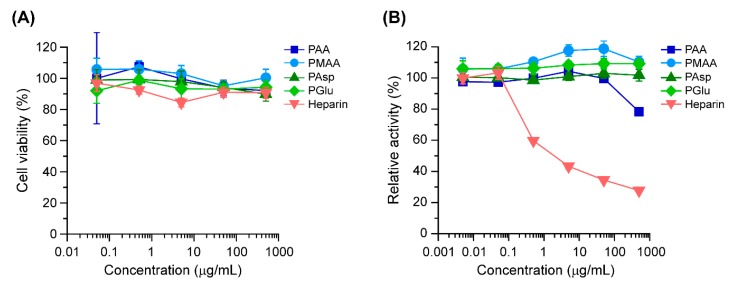
(**A**) Relative viability of MC3T3-E1 cells treated with polycarboxylates and heparin at various concentrations for 24 h (n = 6). (**B**) Relative enzymatic activity of Factor Xa after incubation with a mixture of antithrombin III and polycarboxylates or heparin at various polymer concentrations (n = 3). The plots represent PAA (squares), PMAA (circles), PAsp (triangle), PGlu (diamonds), and heparin (down triangles). The data are expressed as the mean ± standard deviation (SD).

To examine the biocompatibility of polycarboxylates, the cytotoxicity and anticoagulant activity of the polycarboxylates were evaluated. Cytotoxicity was determined in MC3T3-E1 cells after 24 h of treatment with the polycarboxylates. As a result, all the polycarboxylates and heparin showed negligible toxicity in MC3T3-E1 cells at a concentration of 500 μg/mL (Figure 4A).

Heparin has strong anticoagulant effect because it specifically forms a complex with antithrombin III and changes the conformation of antithrombin III [33]. This results in the inhibition of coagulation factors such as Factor IIa (thrombin) and Xa [34,35], leading to anticoagulant effects. Because bleeding can interrupt surgical operation and the healing process after surgery, anticoagulant properties should be avoided in materials. The anticoagulant ability of polycarboxylates was investigated with regard to the enzymatic activity of Factor Xa after incubation with a mixture of polycarboxylates and antithrombin III. As a consequence, heparin markedly inhibited the enzymatic activity of Factor Xa at a concentration of 0.5 μg/mL (Figure 4B). In the case of PMAA, PAsp, and PGlu, the enzymatic activity of Factor Xa remained unchanged even at 500 μg/mL. However, PAA at 500 μg/mL slightly inhibited Factor Xa. Monien reported that PAA can bind to antithrombin and accelerate the inhabitation of Factor IIa and Xa [36]. Although the inhibitory effect of antithrombin III by PAA was significantly lower than that of heparin, it is considered that PAA exerts potent anticoagulant effects.

### 3.3. ALP Activity in MC3T3-E1 Cells Treated with the Polycarboxylate/BMP-2 Complexes

**Figure 5:**
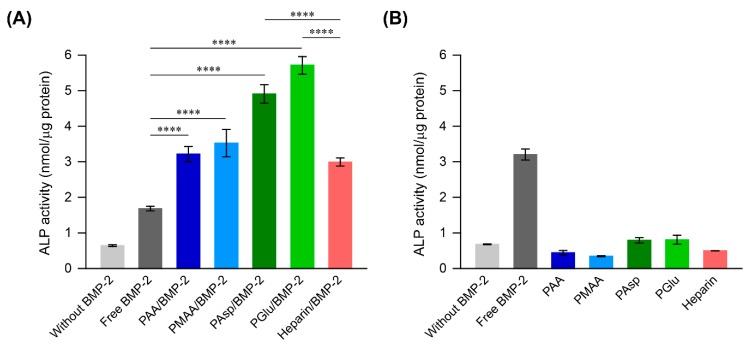
(**A**) Alkaline phosphatase (ALP) activity in MC3T3-E1 cells after 72 h of treatment with free BMP-2, polycarboxylate/BMP-2 complexes, and heparin/BMP-2 complex at polymer and BMP-2 concentrations of 500 μg/mL and 50 ng/mL, respectively (n = 3 in a single experiment). (**B**) ALP activity in MC3T3-E1 cells after 72 h of treatment with free BMP-2 (50 ng/mL), polycarboxylates (500 μg/mL), and heparin (500 μg/mL) (n = 3 in a single experiment). The data are expressed as the mean ± SD (**** *p* < 0.001).

ALP activity is known as an important early stage marker of osteogenic differentiation. Lee described that the activation of the BMP-2 signaling pathway by BMP-2 treatment or by overexpression of BMP-2 receptors strongly stimulates ALP activity [3]. Therefore, ALP activity in MC3T3-E1 cells was evaluated to verify the early stage osteogenic differentiation ability of polycarboxylate/BMP-2 complexes. In these experiments, the concentration of BMP-2 was adjusted to 50 ng/mL because treatment of cells with 50 ng/mL BMP-2 significantly increased ALP activity compared with that in untreated cells (data not shown). When each polycarboxylate was added to BMP-2 at the concentration of 500 μg/mL, the ALP activity in MC3T3-E1 cells treated with each polycarboxylate/BMP-2 complex increased significantly compared to free BMP-2 (Figure 5A). In this experiment, the heparin/BMP-2 complex was also tested as a control because of its ability to enhance osteogenic differentiation activity of BMP-2 [19,20,21]. Consistent with previous studies, heparin/BMP-2 complex induced significantly higher ALP activity than that of cells treated with free BMP-2 (Figure 5A). Interestingly, the cells treated with PAsp/BMP-2 and PGlu/BMP-2 complexes resulted in higher ALP activity than the heparin/BMP-2 complex. Especially, the PGlu/BMP-2 complex led to significantly high ALP activity compared to the other complexes. The negatively charged polymers such as heparin and sulfonated polymers can form a polyelectrolyte complex with BMP-2 and protect BMP-2 from the deactivation such as the interaction with Noggin [20]. We have previously confirmed that the sulfonated polyrotaxanes attenuate the deactivation of BMP-2 in the presence of Noggin [6]. Therefore, it is considered that the polycarboxylates also protect the deactivation of BMP-2, resulting in enhancing the ALP activity of MC3T3-E1 cells.

Figure 5B shows the ALP activity of MC3T3-E1 cells treated with only polycarboxylates or heparin. When the cells were treated with polymers only, the ALP activity were remained comparable to that of cells cultured without BMP-2. This result suggested that the enhanced ALP activity induced by the polycarboxylate/BMP-2 complexes was not attributed to the polymers, and the complexation between the polycarboxylates and BMP-2 contributed to enhancing the osteogenic differentiation activity of BMP-2.

### 3.4. Mineralization of MC3T3-E1 Cells Treated with Polycarboxylate/BMP-2 Complexes

**Figure 6:**
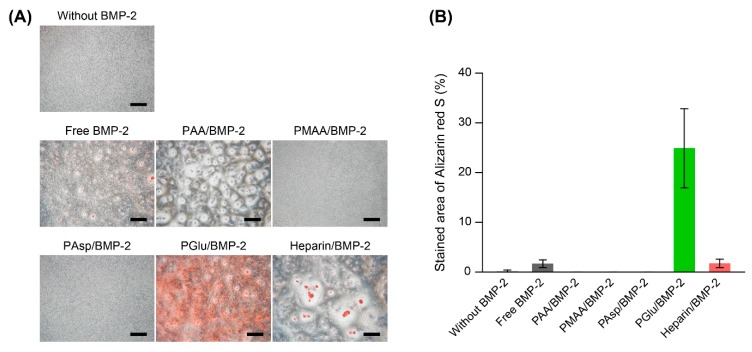
(**A**) Alizarin red S staining of MC3T3-E1 cells treated with free BMP-2, PAA/BMP-2, PMAA/BMP-2, PAsp/BMP-2, PGlu/BMP-2, and heparin/BMP-2 complexes for 14 d (Scale bars: 500 μm). (**B**) Relative area of MC3T3-E1 cells stained with Alizarin red S (n = 4). The concentrations of BMP-2 and the polymers were 100 ng/mL and 1000 μg/mL, respectively.

Because cell mineralization is a strongly evidence of osteogenic differentiation, the deposited mineralized matrix in MC3T3-E1 cells treated with free BMP-2 or polycarboxylate/BMP-2 complexes was stained with Alizarin red S, which specifically binds to calcium in the mineralized matrix (Figure 6A) [37,38]. Untreated MC3T3-E1 cells, which were cultured in osteogenic differentiation medium without BMP-2, did not show mineralized matrix deposition at 14 d. Cells cultured in osteogenic differentiation medium containing free BMP-2 were slightly stained with Alizarin red S. It is considered that the cells were not stained because in these conditions culture period was short (generally, 21 to 28 d of culture is required). In the case of the polycarboxylate/BMP-2, remarkable staining was observed in PGlu/BMP-2 complex-treated cells (Figure 6A). Image analysis of stained area of Alizarin red also supports the suggestion that the mineralization of MC3T3-E1 cells was induced by PGlu/BMP-2 complexes at 14 d of culture (Figure 6B). However, these results were inconsistent with the results of ALP activity (Figure 5A). Although the all polycarboxylates can enhance the activity of BMP-2 at the early stage of osteogenic differentiation, it is considered that the effect is not prolonged until the mineralization of the cells. Accordingly, PGlu is considered as a potential candidate for enhancing the osteoinductivity of BMP-2. However, we could not clarify why PGlu show superior effects in modulating the activity of BMP-2. The ALP activity (Figure 5A) and mineralization (Figure 6) in MC3T3-E1 cells were not correlated with the properties of polymers such as the *M*_n,SEC_ or p*K*_a_ of the polymers (Table 1). It is considered that the chemical structure of PGlu is suitable to form a complex with BMP-2 and protect BMP-2 from the deactivation.

## 4. Conclusions

In summary, a series of four polycarboxylates were demonstrated for enhancing the osteoinductivity of BMP-2. PMAA, PAsp, and PGlu showed no toxicity and anticoagulant activity, while PAA exhibited weak anticoagulant activity at high concentration. The capability of the polycarboxylates to enhance the osteogenic activity of BMP-2 was evaluated by measuring ALP activity and mineralization of MC3T3-E1 cells. The PGlu/BMP-2 complex induced the highest ALP activity and most rapid mineralization compared to the heparin/BMP-2 complex. Consequently, PGlu is a promising candidate for enhancing the osteoinductivity of BMP-2 and could serve as an excipient for BMP-2 or a building block for biomaterials for bone regeneration.

## Figures and Tables

**Table 1:** Characterization of polycarboxylates and heparin.

Sample	*M*_n,SEC_^1^	*M*_w_/*M*_n_^1^	p*K*_a_^2^	α at pH 7.4 ^2^
PAA	10,100	1.56	6.65	0.71
PMAA	5100	1.36	6.84	0.64
PAsp	13,500	1.37	5.74	0.94
PGlu	12,700	1.16	6.05	0.90
Heparin	25,600	1.23	-	-

^1^ Determined by SEC eluted with 100 mM NaNO_3_ at 40 °C. The relative number-average molecular weight of the polymers was calculated based on a calibration curve of standard poly(ethylene glycol)s. ^2^ Determined from pH-α curves.
